# City Mortality

**Published:** 1853-10

**Authors:** 


From the New Orleans Monthly Medical Re_ister.

*City Mortality.*

In another page will be found our record of the mortality for the year ending August 30th.* The large increase of deaths over that of the previous year is startling, and chiefly confined to our class of Zymotic diseases, among which Fevers, and, pre-eminently Yellow fever, exhibit the most marked gain. Another feature is obvious on comparing our tables of the year just closed with its predecessor, in the wide difference exhibited by our endemic yellow fever. While we record in the latter 10 deaths from this fever for July, and 68 for August, we, for the same months of the present year, show an increase beyond, we had almost said, the power of figures to express—an increase, at least so astounding, as to fix the attention with a view to the elucidation of the causes of this enormous difference. Why is it that in '52, on the same soil, among the same population, under the same climatic influences, the same disease, felt in its most benign forms and scarcely attracting notice among the current events of the passing hour, becomes in '53, a deadly pastilence, scattering death, dismay and suffering among our affrighted population? What is it that has so changed the character and increased the fatal influence of this terrible scourge? The true and satisfactory solution of this question involves to a greater degree than is generally believed, the future interests and well-doing of this community, while it offers a point of purely professional interest, which we trust to see fully investigated and determined. There must be some great and powerful influences in operation to produce results so unequal and contrasted. What these are and what their origin and mode of action become to us, under the present circumstances, scarce yet recovered from the shock of such appaling mortality as we have lately witnessed, matters of general and momentous interest. They surpass in the importance of their claims on the intelligence and humanity of our citizens, on the spirit and devotion of our public authorities, and on the skill and knowledge of the medical profession, all other matters of public or general concernment. For it must be obvious, even to the most casual observation, that unless the salubriousness of our city keeps pace with the efforts making to widen its points of contact with our interior country, to multiply its resources of trade and to augment its wealth and industry, we shall be destined to reap the disastrous fruits of wasted labor and capital, and to see hopes wither, which, in view of the felicitous changes the future was garnering up, reconciled us to the exactions of a heavy and burdensome taxation.

*Total deaths during the month of August, 5707.—Yellow Fever, 47[illegible]8. Returns still incomplete.

These sacrifices will all have been incurred in vain, if the future is to be rendered uncertain by the recurrence of such cruel visitations of disease, and life and health hazarded in so inhospitable a clime. So apparent is all this, that we cannot see how the capitalist who has risked his money in investments, or the merchant who has effected his plans for the demands and supply of trade, or the laborer who counts on making his labor and time valuable, or the professional man who finds the true theatre for his skill and learning in an active, busy and progressing community, can fail to discover in our late disastrous affliction a common and overshadowing evil adverse to the hopes and calculations of each, and appealing to a common sense of self-security for its abatement. Emphatically at such junctures in the history of social bodies do questions relating to their physical health and well-being rise to a magnitude beyond all others, claiming consideration at the hands of the wise and philanthropic. We hope, therefore, to see this matter so pressed on the public attention, as ere long by the anxieties it will awaken, and the enquiries it will cause to be instituted, we shall be better prepared to encounter another summer solstice with assurances that will give quiet to apprehension, and such guarantees to the public health, as are within the province of a high moral probability and the deductions of a rational science. Until something like this be done, until the origin of the late pestilence is fully and fairly traced, and its line of march and mode of diffusion determined upon such evidence as would satisfy a candid and unbiassed mind, all hasty and precipitate action should be deprecated. Opinions formed under the excitement of great public distress are too apt to be partial and defective. When the causes of a destructive pestilence, and even its essential nature lie so deep and beyond the general experience of those best informed in matters of this nature, it would be the last act of folly on behalf of the authorities to institute measures of prevention or relief that may prove inadequate or unsuited to the end sought. And here occur the first and main points to be settled in our investigations of this subject; viz.: what is yellow fever? and secondly is it contagious? We are not going into a discussion at present of either of these issues. Neither our space or inclination, in the absence of all the main facts touching the late pestilence, will warrant this. We are free to admit, however, that there is much to be said, and cogently and logically said, on points that have heretofore been regarded as the *res adjudicata*, the settled doctrines of the profession thereon. If at a blush, and confining our view only to the phenomena we have just witnessed of its origin, (such as is commonly believed, but which needs the most thorough scrutiny, in our judgment, before it is accepted), and its modes of spread in our city and those places in daily and direct communication with it, we should venture the notion of its being something beside, something in addition to our ordinary form of endemic yellow fever, and that in diffusing itself slowly and surely along the common routes of travel and intercourse, it claims alliance to contagious disorders; we should hardly do violence to the truth of first impressions. Yet the question returns upon us, if this be so, wherein lies the difference between it and our endemic yellow fever, which, in the months of July, August, September, October and November last, numbered only 464 victims, which showed no power of self-multiplication, nor agency in its spread that could suggest any property in common with contagious disorders. Both forms commenced alike, run their course alike, and terminated alike—both presented the same symptomatology and the same morbid results, death with black vomit and yellowness of the skin—sensible changes in the condition of the body, which suppose a common pathology for both. Have we not encountered here, at the threshold as it were, a difficulty which can only be overcome by patient investigation and by impartial sifting of all evidence relating to the subject. If they be different diseases, in what consists this difference? Surely not in the fact of a difference in origin, or manner of diffusion, or both. This would be assuming as true the very point at issue—on error in logic, but too common with those who reason from a partial and limited number of facts. Is any one prepared to show the origin of the late pestilence, to give us the history of the first case, its communication with an admitted source of infection, and the spread from it as a centre, of its deadly energies throughout our community, our State, our entire Gulf Coast, from the shores of Florida to the bays and harbors of Texas? If after all this has been done, can the next step in the proof of its distinctive character, and its possession of a contagious property, be as satisfactorily determined—viz.: to show an authentic example of its having been communicated by the transmission, mediately or immediately, of a virus derived from the diseased, by inoculation with the blood, or with the morbid secretions from the fluids?

Yet all this is essentially required by the defined and accurate professional judgments of the day, in order to meet the condition of diseases in themselves contagious and as contra-distinguished from epidemic disorders. Again, in becoming so wide-spread, so literally and truly epidemial as we observe of the late pestilence, are there not involved in the very terms themselves, conditions external to the disease, conditions of atmosphere favorable to its diffusion, and without which the disease would assuredly cease. How else and with what seeming propriety can we limit its duration to periods of time marked by high temperature, or recognize the power of cold or violent atmospheric commotions to arrest it? If the virus exists and it be thus subject to atmospheric states for its very powers of increase and spread, are we not met by difficulties greater and more obscure than that which concerns the proof of its contagion? Obstacles of this nature meets us at every step and suggest the wisdom of duly weighing every tittle of evidence that may be brought to bear on its investigation. We have thrown out these detached observations in the hope of eliciting a full and ample statement of all that may be positively known by any of our citizens bearing on this matter. It should be calmly and sophistically studied as a great question of social economy, affecting the arrangements and relations of society in their most extended sense. It concerns directly the present happiness and moral being of every individual of us and remotely and in the future, the destinies and welfare of our children's children.

As a purely professional question we have no cheering or abiding hopes that it will be discussed in a spirit so as to ensure harmony and uniformity of opinion among the votaries of the profession. The nature of medical evidence is such as to forbid this. But whatever may be the differences of opinion, it is but proper we should have all the facts pertaining to this issue. To do this fully and satisfactorily, our public authorities should institute a Commission of competent persons, to collect all the incidents of its recent outbreak. It should be authorized to summon witnesses and to compel attendance, just as in matters of preliminary investigation, before a committing magistrate. It is essential that the whole truth be known, if it be desirable to base on the results of the investigation, measures at once novel and contradictory to our past usages and experience. Wise and proper as this caution may be, however, it must be borne in mind that duties of a character altogether different devolve upon us. If it should be determined upon ample and accurate evidence that the peculiar virus of yellow fever is something transportable with the body of the sick, or with his clothes, or through the medium of merchandise, or in any tangible shape whatsoever, it must not be forgotten that the virus must find accessaries in the localities of communities, in their meteorological states, and in the susceptibility of their population in order to multiply and diffuse itself. Carried to latitudes beyond its prescribed and accustomed geographical limits, and it dies out or becomes inert and inoperative. This is but too apparent to all who are conversant with the history of the pestilence—in fact it is but one example of what seems to be a law of nature in regard to epidemic disorders. We should no more expect to see yellow fever prevail in high and northern latitudes, than to see typhus rage in intertropical lines, the cold of the one like the heat of the other region, at once and effectually extinguishing them. Our enquiries may then be said to have only begun when we shall have ascertained that we owe to a foreign source the origin of our pestilence. We must turn our eyes inward and learn if a sanatory police cannot be made useful in the removal of offals and the general filth, common to large cities—in the institution of ordinances, regulating the manner of draining and filling vacant lots, of paving streets, providing ample and pure supplies of water, of cleansing privies, of building shanties, the destined abode of our poor and needy population, and of closing the wretched rookeries, whose every hole and corner is crowded with human beings, to a degree and manner shocking to every sense of decency and propriety, and alarming from the gross infraction of the most essential rules of health. No one can deny that duties of this kind are within the province of our governing authorities. We have a fruitful element of disease annually accumulating in our midst, in the growth and increase of our foreign population. They bring with them not only bodies susceptible by their foreign birth to our endemial disorders, but habits and customs, as unlike and unsuited to our climate and usages. They come from wretched and crowded hovels, where want and filth produce pestilence, to our cities and towns, where they cluster in numbers as thick and live amid filth as gross as that they have escaped from. They come to find employment and ready remuneration for their labor, and they live like persons just released from the pains of famine. They eat and drink to excess. They violate by day and night every maxim of prudence, every safeguard of health. Surely this is most serious matter for consideration, for amendment, for reform. The fault may not be theirs—poverty and oppression at home may have caused much of this huge evil. They know no better. All the traditions of home and family record no variety to their woes. It was want, and privation, and suffering and filth before their day. It is the heritage they derive from their parents and friends—it is the sole accompaniment, the invariable attendants upon them in their pilgrimage to our shores. We must, therefore, look to their domestic relations, we must subject their social irregularities to control and discipline, if we wish to do them good service, and to exempt ourselves from the destroying ravages of a cruel pestilence. They must be taught to value not only the blessings of political freedom, which they gain by coming to our shores, but to learn how to value the higher blessings and comforts of a good, well ordered and salubrious home. One means to insure this will be to discourage by stringent laws the habit of sub-leases to tenants, which leads to overcrowding and to all the subsequent ills which attend on this. This is become too common an abuse of property among that class. A landlord rents his house and lot to one person, who sub-leases to a dozen or more families, the more the better for the original lessee—no matter what abuses result therefrom, and how the general and other interests are made to suffer. And generally, too, it is the old and decaying property, whose rafters are undermined by time and grown green with mould, that thus falls into the occupancy of this class. As long as it continues decent, or comfortable, or safe, they are excluded by high rents, and a better class from its use. But let it wear by time and neglect till it totters, let it grow dank and unwholesome, let it become but little less than the sheds which house our cattle, and then it becomes the fit habitation for that portion of our population, who are content with all these discomforts, and who seek shelter there as naturally as bats do crannies and dark holes. But enough have we said on this topic. It makes the heart sick to witness the great suffering among this unhappy class. The neglect of society, the indifference of our laws, the aversion of our people to them, conspire with their disorganized condition and mischievous habits to keep perpetual the elements requisite to give malignancy to disease and facility to its spread. But there are other mischievous and hurtful usages which we tolerate apart from our foreign population. We have space to allude to but one, and that a huge and monstrous one. This is the manner in which our city authorities sanction the filling up of the land reclaiming by the changes of our river bed. A viler compost, one more abounding in disgusting offensive nuisances cannot be found anywhere. Standing on an evening after sunset, on any portion of our levee, one might realize something of the disgust of Coleridge at Cologne:

> "He might count two-and-seventy stenches,
> All well-defined and genuine stinks,"

so thick and reeking are the odors escaping from those foul spots. They are the burial places of all dead animals, from a mouse to a horse, the common receptacle of the offals from every cook-shop and kitchen, of the refuse vegetables, bones and garbage of our market houses, and the sweepings of our streets. If the art of man could contrive anything worse than this we should like to see it. Yet we breathe this foul air, worse than the abattoirs of Paris, and wonder that we sicken and die. Rouse up we must and set our household in order, if the future is to be spanned with brighter hopes and stronger assurances. We will have to look more intently at home, more closely into our domestic habits, more narrowly into our social vices, more determinedly on the negligence of our laws, if we are to be any thing beside the immense lazar-house the late pestilence has made us.

